# Synovium Fragment-Derived Cells Exhibit Characteristics Similar to Those of Dissociated Multipotent Cells in Synovial Fluid of the Temporomandibular Joint

**DOI:** 10.1371/journal.pone.0101896

**Published:** 2014-07-08

**Authors:** Yang-peng Sun, You-hua Zheng, Wen-jing Liu, Yu-liang Zheng, Zhi-guang Zhang

**Affiliations:** Department of Oral and Maxillofacial Surgery, Guanghua School of Stomatology, Hospital of Stomatology, Sun Yat-sen University, Guangdong Provincial Key Laboratory of Stomatology, Guangzhou, Guangdong, People's Republic of China; University of Southern California, United States of America

## Abstract

Multipotent mesenchymal stem cells (MSCs) found in the synovial fluid (SFMSCs) of the tempromandibular joint (TMJ) remain poorly understood. During TMJ arthrocentesis, we discovered that synovial fluid collected from some patients with TMJ disorders contained not only SFMSCs but also synovium fragments (SFs). In this study, we attempted to characterize both the SFMSCs and SF-derived cells (SFCs) in order to further understand the role of MSCs in the synovial fluid of the TMJ. The SFs were membranous and translucent and consisted of several cell layers, indicating that their origin was only from the intima. SFCs were obtained by digestion of the SFs and subsequently expanded *in vitro*. SFMSCs were enriched by centrifugation of the synovial fluid and expanded *in vitro*. SFCs and SFMSCs displayed a similar fibroblast-like, spindle-shaped morphology, and we observed that some SFMSCs grew out of small tissue masses in culture. Flow cytometric analysis showed that both groups of cells expressed similar surface markers, including CD90, CD44, CD105, and CD73. However, both were negative for Stro-1, CD146, CD45, CD34, CD11b, CD19, and HLA-DR. Immunofluorescent staining showed that both SFs and SFMSCs expressed vascular cell adhesion molecule 1. Both SFCs and SFMSCs could be induced to differentiate down osteogenic, chondrogenic, adipogenic, and neurogenic lineages *in vitro*. Together, our results indicate that the intima is the most likely tissue origin of SFMSCs in the TMJ. Moreover, the SFs are composed of only intima and thus offer an improved source of synovium-derived MSCs compared to synovium specimens obtained by surgery, which contain both intima and subintima.

## Introduction

Osteoarthritis is one of the most common and serious tempromandibular joint disorders (TMDs). Osteoarthritis is a degenerative joint disease that is accompanied by a progressive reduction in extracellular matrix in joint cartilage and bone that eventually leads to loss of joint function[1]. Due to the limited capacity for self-repair and remodeling of joint tissues, treatment strategies are ineffective and restricted to relieving the symptoms. Such strategies involve different surgical procedures including debridement. Adult mesenchymal stem cells (MSCs), which have the abilities of self-renewal and multipotential differentiation, including chondrogenic differentiation, are considered a promising cell type for cell-based strategies for articular cartilage repair[2]–[7]. MSCs can be obtained from a variety of adult tissues, including synovium and synovial fluid[8]–[11].

Synovial fluid plays an important role in maintaining the normal physiological function of joints[12]. In recent years, many studies have investigated synovial fluid-derived mesenchymal stem cells (SFMSCs), primarily focusing on the knee joint. First, Jones et al.[9] demonstrated the presence of SFMSCs in swollen osteoarthritic knee joints. Then, Harvanova et al.[13] confirmed that cells isolated from synovial membrane specimens and synovial fluid from knee joints express similar cell surface markers. Lee et al.[8], [14] reported that SFMSCs levels are linked with the severity of knee osteoarthritis. Morito et al.[10] reported that the number of SFMSCs increases after intra-articular ligament injury in humans. Together, the results of these studies indicate that SFMSCs are closely associated with osteoarthrosis in the knee joint. However, the role and tissue origin of SFMSCs remain poorly understood.

Fewer studies have investigated the presence and role of SFMSCs in the tempromandibular joint (TMJ). Koyama et al.[11] published the first and only report showing that Stro-1^+^CD146^+^ SFMSCs exist in the synovial fluid of the TMJ in TMD patients. They proposed that these SFMSCs are similar to bone marrow-derived MSCs and also might be a promising candidate cell type for cell-based strategies for articular cartilage repair.

We discovered the presence of synovium fragments (SFs) in synovial fluid collected from some TMD patients during arthrocentesis of the TMJ, and in the present study, we investigated both SF-derived cells (SFCs) and SFMSCs isolated from the TMJ in order to further understand the role of SFMSCs in TMDs. We isolated and expanded both cell types from the synovial fluid of the TMJ collected from TMD patients and then compared their characteristics including *in vitro* proliferation and morphology, surface antigen expression, and multilineage differentiation capabilities.

## Materials and Methods

### Ethics statements

The study protocol was approved by the institutional ethics board of the Hospital of Stomatology, Sun Yat-sen University, Guangzhou, China. All patients had provided written informed consent to participate in this study.

### Synovial fluid dilution and SF collection from the TMJ

A total of 36 diluted synovial fluid samples were used in this study after collection from 29 patients with TMDs that did not respond to conservative treatment. Samples were collected during arthrocentesis of the TMJ. The upper joint compartment was expanded with 1.5–2.0 ml lidocaine using a #8 needle and syringe for local anesthesia, and the fluid was then withdrawn. The 29 patients had no other systemic diseases, and their ages ranged from 18–61 years old. Among these patients, 4 were men and 25 were women. Samples collected from nine patients contained macroscopically visible SFs, and of these patients, 1 was a man and 8 were women. Although samples from only 29 patients were used for this study, in our clinic, we have collected samples from 450 patients (age range, 17–64 years), and samples from only 9 of those patients contained SFs. Thus, the frequency of the presence of SFs in diluted synovial fluid samples was 2% in our clinical experience. The specimens were stored at 4°C within 4 hours of collection.

### Culture of human SFCs

The macroscopic SFs were isolated from the diluted synovial fluid samples individually and washed three times with phosphate-buffered saline (PBS) in sterile Petri dishes. The fragments were then digested with 2.5 ml of 4 mg/ml type I collagenase (Gibco) for 2.5 hours at 37°C. The SFs were dispersed by pipetting until they were no longer visible. Cells were centrifuged at 450 g for 5 min, and after centrifugation, the supernatant was discarded. The cells were washed with PBS once and then centrifuged again. After the supernatant was discarded, the cells were plated in T25 flasks (Corning) with 3 ml complete culture medium [alpha minimum essential medium (α-MEM; Gibco) supplemented with 10% fetal bovine serum (Gibco)]. The cells were expanded in monolayer culture. Portions of the cells from each sample were mixed and expanded in monolayer culture for further experimentation. The remaining synovial fluid dilutions were used for collecting SFMSCs.

### Culture of human SFMSCs

The collected synovial fluid samples were centrifuged at 300 g for 5 min. After centrifugation, the supernatant was discarded, and the cells of individual samples were plated on 10-cm culture dishes (Corning) with 9 ml complete culture medium and incubated at 37°C in 5% CO_2_. After 48 hours, the medium was withdrawn to remove non-adherent cells and replaced with fresh medium. During the first 2 days, the culture dishes were observed closely to monitor the status of adherent cells. The cells were expanded in a monolayer culture. In addition, portions of the cells from each sample were mixed and expanded in monolayer culture for further experimentation.

### Cell proliferation

SFMSCs (passage 4) and SFCs (passage 4) were seeded in 96-well plates at a density of 5,000–10,000 cells/well. The culture medium was changed every other day. Cell proliferation was measured on days 0, 1, 3, and 5 using a Cell Counting Kit-8 (Yeasen). Briefly, the cells were incubated with the Cell Counting Kit-8 reagent in the dark for 1 hour at 37°C. Culture medium combined with Cell Counting Kit-8 reagent served as a blank control. Following the incubation, the optical density (OD) of the solution in each well was measured at 450 nm using a microplate reader (Infinite200, Tecan). Repeated measurements (n = 3) were carried out for each time point. To assess population doubling (PD) and calculate the doubling time (DT), OD values and culture times were measured and recorded. PD and DT were calculated using the following formula: PD = (lnN-lnN0)/ln2, DT = T/PD (N: OD_cells_-OD_blank control_ at end point, N0: OD_cells_-OD_blank control_ at initial time, T: time interval).

### Surface antigen expression profile

Flow cytometry was performed with an FC 500 flow cytometer (Beckman Coulter). The following antibodies were used: Peridinin Chlorophyll protein (PerCP)-Cy5.5-conjugated anti-human CD105 (1∶20; BD Biosciences), allophycocyanin-conjugated anti-human CD73 (1∶20; BD Biosciences), fluorescein isothiocyanate-conjugated anti-human CD90 (1∶20; BD Biosciences), phycoerythrin-conjugated anti-human CD44 (1∶20; BD Biosciences), phycoerythrin-conjugated anti-human CD45/CD34 CD11b/CD19/HLA-DR (1∶20; BD Biosciences), fluorescein isothiocyanate-conjugated anti-human Stro-1 (1∶20; Biolegend), and phycoerythrin-conjugated anti-human CD146 (1∶5; BD Biosciences). The cells were digested in culture flasks or dishes with 0.25% trypsin (Gibco) for 2 min before the digestion reaction was neutralized with complete culture medium. The cells were centrifuged at 300 g for 8 min, and the supernatant was discarded. The cells were resuspended in PBS, transferred to 1.5-ml Eppendorf tubes (approximately 5×10^5^ cells per tube), and centrifuged again at 300 g for 8 min. The supernatant was discarded, and the cells were resuspended in PBS and incubated with the antibodies or negative control antibodies for 30 min at 4°C at a total volume of 100 µl per tube. After incubation with antibodies, the cells were centrifuged at 300 g for 8 min, and the supernatant was discarded. The cells were resuspended in 0.8 ml PBS, and flow cytometry analysis using MXP Software (Beckman Coulter) was conducted according to the manufacturer's instructions.

### Immunofluorescent staining for vascular cell adhesion molecule 1 (VCAM-1)

The cells were plated in 35-mm glass bottom dishes (MatTek) and incubated in complete culture medium at 37°C in 5% CO_2_. At approximately 50% confluence, the medium was aspirated, and the cells were washed twice with PBS. The cells were then fixed in 4% paraformaldehyde for 30 min before treatment with 1X PBS-0.3% Triton-X100 for 15 min at room temperature. The cells were blocked for 2 hours with 1 ml of 1X PBS-5% bovine serum albumin (BSA). After two 5-min washes with 1 ml of 1X PBS, the cells were stained with rabbit anti-human VCAM-1 (Santa Cruz Biotechnology) diluted at 1∶100 in 1X PBS-1% BSA for 18 hours at 4°C. After three 5-min washes with 1X PBS, the cells were incubated with the secondary antibody, dylight 488-TFP ester-conjugated goat anti-rabbit IgG antibody (EarthOx) diluted 1∶100 in 1X PBS-1% BSA for 60 min at 37°C. After three 5-min washes with 1X PBS, the cells were incubated with 1 µg/ml DAPI (Cell Signaling Technology) for 5 min. The cells were then washed three final times with 1X PBS for 5 min before viewing under confocal microscopy (Zeiss). Cells were treated using the same process, but without incubation with a primary antibody as control.

### Differentiation and evaluation of isolated SFCs and SFMSCs

#### Osteogenic differentiation

The cells were plated in 6-well plates and incubated in complete culture medium at 37°C in 5% CO_2_. At approximately 40–50% confluence, the medium was replaced with a previously reported osteogenic induction medium that consisted of α-MEM (Gibco) containing 10% FBS (Gibco), 10 mM sodium β-glycerophosphate (Santa Cruz Biotechnology), 100 nM dexamethasone (MP Biomedicals), and 50 µM ascorbic acid-2-phosphate (Wako)[15]. The control group was incubated in complete culture medium, and for both groups, the medium was replaced every 3 days for 28 days.

Alkaline phosphatase activity was assessed after 14 days in osteogenic differentiation medium. The growth medium was removed and the cells were washed with PBS and lysed in radioimmunoprecipitation assay (RIPA) lysis buffer (Beyotime) supplemented with 1% phenylmethylsulfonyl fluoride (PMSF; Beyotime). The protein concentration was determined using a BCA Protein Assay Kit (Beyotime). ALP activity was determined using an ALP microplate test kit (Nangjing Jiancheng Bioengineering Institute, China).

Osteogenesis after 28 days in induction medium was assessed by alizarin red staining and Von Kossa staining. For alizarin red staining, the cells were rinsed with PBS, fixed in 4% paraformaldehyde for 10 min, rinsed twice with distilled water, and stained with a fresh 0.1% alizarin red solution for 30 min at 37°C. The cells were then washed twice with distilled water and examined under an inverted phase contrast microscope (Axiovert 40, Zeiss). For Von Kossa staining, a Von Kossa staining kit (GenMed Scientifics) was applied according to the manufacturer's guidelines.

#### Adipogenic differentiation

The cells were plated in 6-well plates and cultured in complete culture medium. At approximately 50% confluence, the medium was replaced with a previously reported adipogenic induction medium that consisted of α-MEM (Gibco) containing 10% FBS (Gibco), 200 µM indomethacin (Sigma-Aldrich), 0.5 mM isobutyl methylxanthin (MP Biomedicals), 1 µM dexamethasone (MP Biomedicals), and 10 µg/ml insulin (MP Biomedicals)[15]. The control group was incubated in complete culture medium, and for both groups, the medium was replaced every 3 days for 28 days.

After adipogenic induction for 28 days, the cells were rinsed twice with PBS, fixed in 4% paraformaldehyde for 10 min, again rinsed twice with PBS, stained with a fresh 0.3% oil red O solution and a fresh 0.5% sudan black B solution individually for 150–180 seconds, and washed with 70% ethanol slightly until the background was clean. Cells were then examined under an inverted phase contrast microscope (Axiovert 40, Zeiss).

#### Neuronal differentiation

The cells were plated in 6-well plates and maintained in complete culture medium at 37°C in 5% CO_2_. After reaching 60–80% confluence, the medium was replaced with a previously reported neuronal induction medium that consisted of Neurobasal-A media (Gibco) containing 1X B-27 Supplement (Gibco), 20 ng/ml recombinant human epidermal growth factor (rhEGF; Peprotech), 40 ng/ml basic fibroblast growth factor (bFGF; Peprotech), 10 ng/ml brain-derived neurotrophic factor (BDNF; Prospec), 1 mM N(6),2'-O-dibutyryladenosine 3':5' cyclic monophosphate (dbCAMP; Sigma-Aldrich), 0.5 mM isobutyl methylxanthin (MP Biomedicals), and 10 ng/ml FGF-8 (Peprotech) for 12 hours to induce differentiation[16]. The control group was incubated in complete culture medium.

Neuronal differentiation after 12 hours in induction medium was assessed by immunofluorescent staining for two neuronal cell markers, nestin and glial fibrillary acidic protein. For nestin immunostaining, mouse anti-human antibody nestin (Santa Cruz Biotechnology) was used and diluted 1∶100. For glial fibrillary acidic protein immunostaining, mouse anti-human antibody to glial fibrillary acidic protein (Millipore) was used and diluted 1∶200. Secondary antibody FITC-conjugated goat anti-mouse IgG antibody (Santa Cruz Biotechnology) was used and diluted 1∶200. The procedures used for immunofluorescent staining were the same as described above.

#### Chondrogenic differentiation

Approximately 2 million cells were transferred into a 15-ml centrifuge tube and then centrifuged at 450 g for 10 min. Then 450 µl chondrocyte differentiation induction medium that consisted of α-MEM (Gibco), 1X ITS-A (Gibco), 100 nM dexamethasone (MP Biomedicals), 50 µM ascorbic acid (Sigma-Aldrich), 40 µg/ml proline (Sigma-Aldrich), and 10 ng/ml transforming growth factor (TGF)-β1 (PeproTech) was added. The medium was refreshed every 3 days for 21 days, taking extra caution to avoid disrupting the cell mass. The control group was incubated with complete culture medium.

After culture in chondrogenic differentiation medium for 21 days, chondrogenic differentiation was assessed by histological staining. Cartilage nodules were fixed with 4% formalin at 4°C for more than 24 hours and then placed in embedding cassettes. The procedure for paraffin embedding was as follows: 70% ethanol, two changes, 1 hour each; 80% ethanol, one change, 1 hour; 95% ethanol, one change, 1 hour; 100% ethanol, three changes, 1.5 hour each; oil of turpentine (TO) clearing reagent (Rosin Factory CenXi City, China), three changes, 1.5 hour each; and paraffin wax at 60°C, two changes, 2 hours each. Once samples were embedded in paraffin blocks, the paraffin blocks were trimmed as necessary and cut at 5 µm. Each paraffin ribbon was placed in a water bath at approximately 40°C. Sections were mounted onto slides, allowed to air dry for 30 min, and then baked in a 45°C oven overnight. Sections were deparaffinized in three changes of TO (10 min each) and then rehydrated in two changes of 100% ethanol for 3 min each, followed by 95% and 80% ethanol for 1 min each, before finally rinsing with distilled water.

The procedure for Safranin O staining was as follows: sections were stained in 0.1% safranin O solution for 5 minutes, dehydrated and cleared with 95% ethanol, 100% ethanol, and TO clearing agent, with 2 changes of each for 2 min each. A drop of permount (TM) mounting medium (Jianglaibio) was placed on each slide using a glass rod, taking care to leave no bubbles before the slides were coverslipped, dried overnight, and observed under a microscope (Axioskop 40, Zeiss).

The procedure for immunocytochemical staining for collagen type II was as follows: sections of cartilage nodule tissue were deparaffinized and rehydrated and then 3% H_2_O_2_ was applied for 10 min at room temperature before two washes with double distilled water (ddH_2_O) for 2 min each. Slides were next incubated in 0.01 M citrate buffer [3 g/l trisodium citrate (C_6_H_5_Na_3_O_7_·2H_2_O) and 0.4 g/l citric acid (C_6_H_8_O_7_·H_2_O) in ddH_2_O] for 20 min at 94–98°C, then cooled at room temperature for 30 min, washed twice in PBS for 2 min each, immersed in blocking buffer (5% BSA in PBS), and incubated at 37°C for 1 hour. The tissue sections were then incubated in a solution of rabbit anti-human antibody to collagen type II (Sigma-Aldrich) diluted 1∶80 in blocking buffer for 16 hours at 4°C. After three 5-min washes in PBS, slides were incubated in a solution of biotinylated goat anti-rabbit IgG (Boster) for 30 min at 37°C. Slides were then washed in PBS, and streptavidin-biotin complex reagent (Boster) was applied and allowed to react for 30 min at 37°C. After three 3-min washes in PBS, 3,3'-diaminobenzidine (DAB; Boster) was applied to visualize the staining, which was viewed under light microscopy (Axioskop 40, Zeiss). Sections were treated using the same process but without incubation with a primary antibody as control.

### Hematoxylin and eosin (HE) staining of SF sections

SFs were fixed in 4% formalin for 48 hours at room temperature and then placed in embedding cassettes. The procedures used for paraffin embedding, section deparaffinization, and rehydration were the same as described above. For HE staining, sections were incubated in Mayer's hematoxylin for 15 minutes, washed under running tap water for 20 min, stained with eosin for 1 min, and dehydrated in 95% and 100% alcohol, with two changes of each for 2 minutes each. Sections were cleared with two 2-min changes of TO clearing agent, mounted using Permount, and observed under light microscopy (Axioskop 40, Zeiss).

### Gene expression analysis by RT-PCR

Total RNA was isolated using TRIzol reagent (Invtrogen) according to the manufacturer's instructions. cDNA was synthesized using the RvertAid First Strand cDNA Synthesis kit (Thermo Scientific). First strand cDNA synthesis was performed by a 5-min incubation at 25°C, then a 60-min incubation at 42°C, and finally a 5-min incubation at 72°C to inactivate the program. Relative mRNA expression levels for the runt-related transcription factor 2, osteocalcin, and lipoprotein lipase genes (*runx-2*, *ocn,* and, *lpl*, respectively) were determined by PCR using *gapdh* as the reference control. The primers for these genes are listed in Table 1.

**Table 1:** Oligonucleotide primers used in RT-PCR.

Gene	Primer sequence	Product size (bp)
*gapdh*	Forward: CAAGGTCATCCATGACAACTTTG	496
	Reverse: GTCCACCACCCTGTTGCTGTAG	
*gapdh*	Forward: ACCCACTCCTCCACCTTTG	178
	Reverse: CTCTTGTGCTCTTGCTGGG	
*ocn*	Forward: CCACCGAGACACCATGAGAG	267
	Reverse: TCAGCCAACTCGTCACAGTC	
*runx-2*	Forward: TCAACGATCTGAGATTTGTGGG	81
	Reverse: GGGGAGGATTTGTGAAGACGG	
*lpl*	Forward: CAAGAGTGAGTGAACAAC	189
	Reverse: AATTATGCTGAAGGACAAC	

Abbreviations: gapdh, glyceraldehyde-3-phosphate dehydrogenase; ocn, osteocalcin; runx-2, runt-related transcription factor 2; lpl, lipoprotein lipase.

PCR was performed using Taq DNA polymerase (TaKaRa premix Tag, version 2.0 plus dye, TaKaRa, Japan) with the following amplification conditions:

For *lpl*, denaturation at 95°C for 3 min, followed by 40 cycles of 95°C for 30 sec, 55°C for 30 sec, 72°C for 45 sec, then extension at 72°C for 10 min, and finally held at 4°C.

For the *gapdh* primer that yielded a 496-bp product as well as for *ocn*, denaturation at 95°C for 3 min, followed by 40 cycles of 95°C for 30 sec, 60°C for 30 sec, 72°C for 45 sec, then extension at 72°C for 10 min, and finally held at 4°C.

For *runx-2* and *the gapdh* primer that yielded a 178-bp product, a SYBR Green qPCR kit was applied (Thermo Scientific, #K0251) with an initial denaturation at 95°C for 2 min, followed by 40 cycles of 95°C for 15 sec and 60°C for 60 sec. Then a melting curve analysis was performed. The 2^-ΔΔCT^ method[17] was used to analyze the relative gene expression levels by normalizing with *gapdh* as an endogenous control and calibrating with efficiency. ΔΔCT was calculated using the following formula: (Ct_target gene_ – Ct*_gapdh_*)_sample_ - (Ct_target gene_ – Ct*_gapdh_*)_control_.

PCR products were subjected to 2% agarose gel (Invitrogen) electrophoresis. To evaluate relative gene transcription levels, a semi-quantitative method was used. ImageJ software (NIH) was used to collect the gray intensity values of the PCR bands, which were normalized by the intensity value for *gapdh* expression in the same sample. Student's t-test was used to analyze differences between the relative gray intensities of the PCR bands, as reported previously[18].

### Statistical analysis

Data were exported to Microsoft Excel for statistical analysis. Results are presented as mean ± standard deviation (SD). The Student's t-test was performed as appropriate. P-values of less than 0.05 were considered significant, and each experiment was repeated at least three times.

## Results

### *In vitro* adherence and morphology of SFMSCs

**Figure 1:**
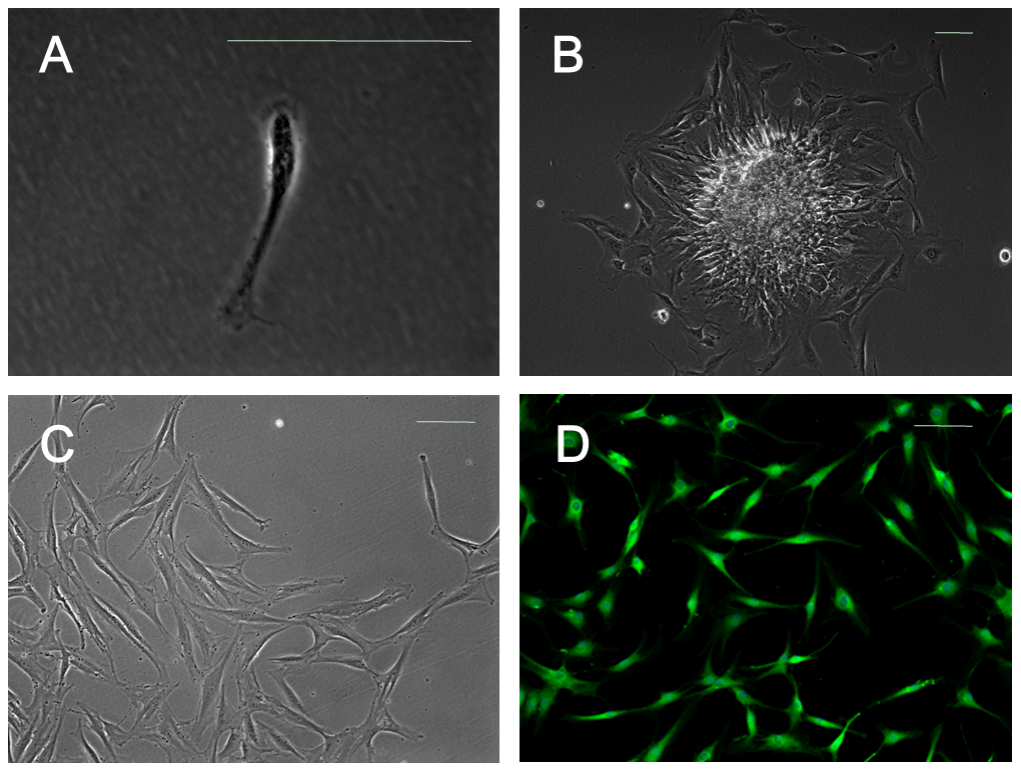
SFMSCs obtained from TMD patients. **(A)** Dissociated single adherent cells were observed after culture of synovial fluid samples for 48**(B)** Cells were observed growing from small tissue masses after culture of synovial fluid samples for 5 days. **(C)** Microscopic image showing the typical morphology of synovial fluid-derived cells. **(D)** Immunofluorescent staining of synovial fluid-derived cells demonstrated positive expression of VCAM-1. Scale bars  =  100 µm.

After plating and culturing the diluted synovial fluid samples for 48 hours, mostly individual cells adhered to the culture plates (Fig. 1A). Cell proliferation was observed after a few days in culture, and the cells exhibited the fibroblastic spindle shape typical of SFMSCs (Fig. 1C). Interestingly, some cells grew out of small tissue masses, which were likely derived from disrupted synovium (Fig. 1B), and this observation is not consistent with the homing theory[9].

### Characterization of SFs and in vitro morphology of SFCs

**Figure 2:**
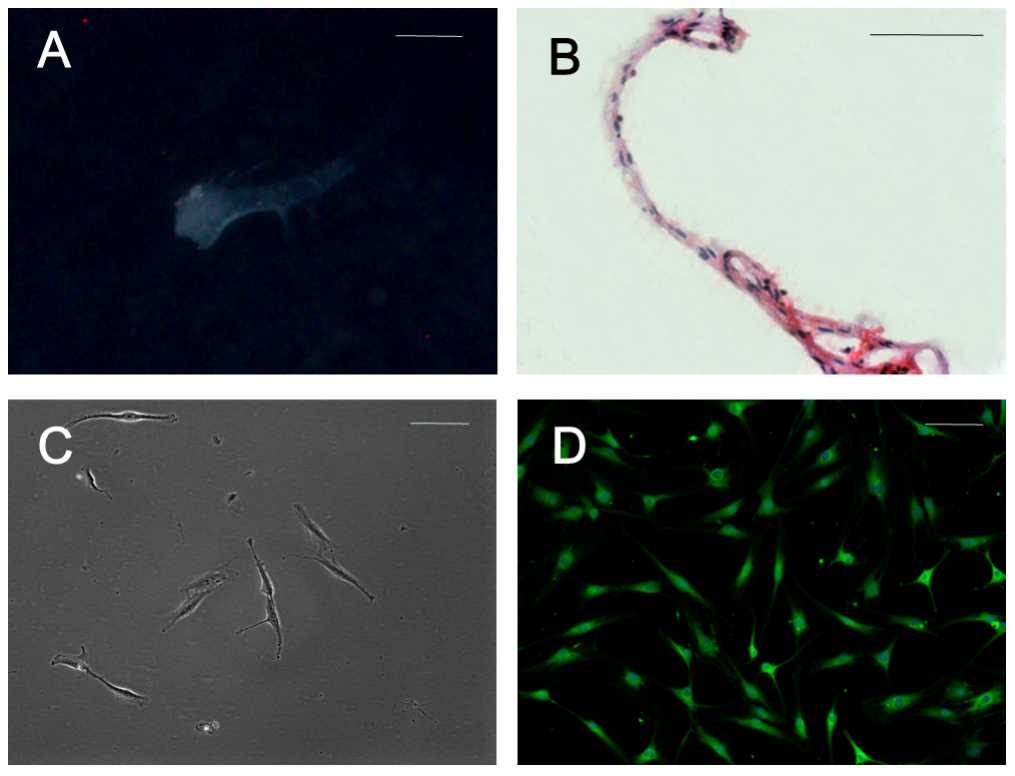
SFs and SFCs obtained from the synovial fluid of TMD patients. **(A)** Gross morphology of a SF under a stereomicroscope (M205A, Leica, Germany). **(B)** An HE-stained section of a SF. **(C)** Typical morphology of SFCs. **(D)** Immunofluorescent staining showed that almost all SFCs were positive for VCAM-1. Scale bar  =  1 mm in (A) and 100 µm in (B-D).

**Figure 3:**
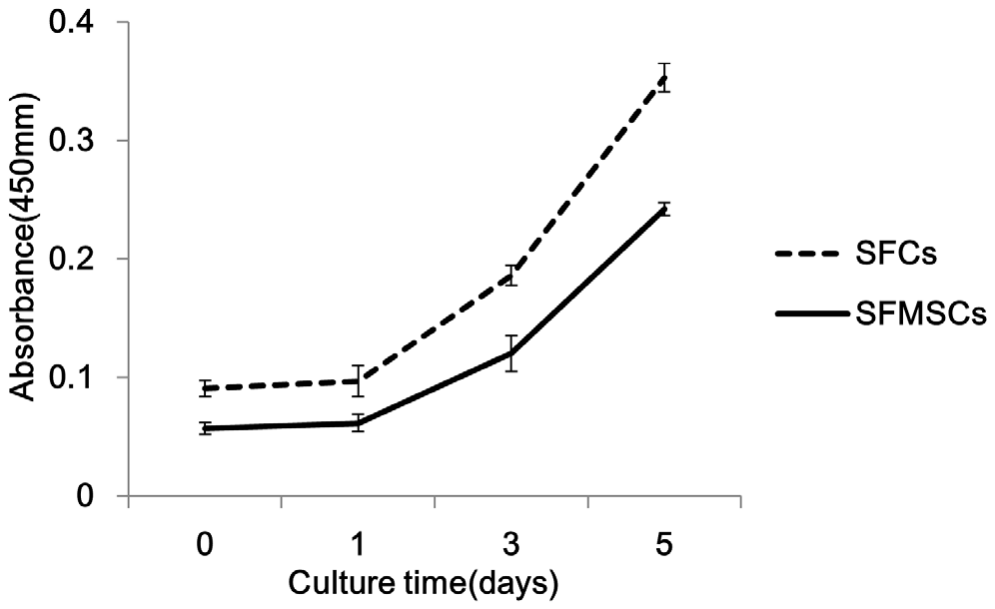
Cell growth curve of SFCs and SFMSCs.

SFs were found in the synovial fluid collected from some TMD patients during arthrocentesis of the TMJ (Fig. 2A). These fragments were membranous and translucent, and histological evaluation showed that these fragments only consisted of multiple layers of cells (Fig. 2B). These characteristics support the hypothesis that the SFs were derived from the intima. After dissociation of the fragments, the resultant SFCs exhibited a fibroblastic spindle shape in culture (Fig. 2C). Cell growth curve showed that cell growth pattern of SFCs was similar to that of SFMSCs (Fig. 3). The average PD of SFMSCs was 1.32±0.47, and SFCs displayed an average PD of 1.25±0.44. The average DT was 48.66±1.00 hours for SFMSCs and 51.42±0.43 hours for SFCs at passage 4.

### Surface antigen expression on SFMSCs and SFCs

**Figure 4:**
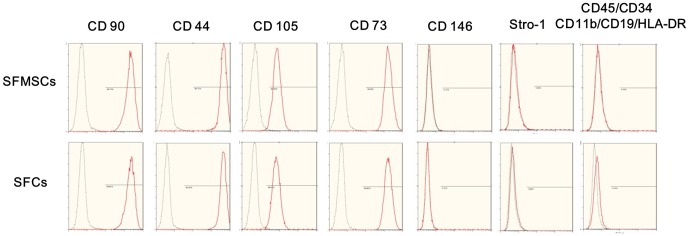
Flow cytometric analysis of SFCs and SFMSCs. Both SFCs and SFMSCs expressed similar surface markers, including CD90, CD44, CD105, and CD73. Stro-1, CD146 CD45, CD34, CD11b, CD19, and HLA-DR were not detected on the surfaces of these cells. Black lines represent negative controls, and red lines are the results for the experimental groups.

We found that almost all *ex vivo*-expanded SFMSCs and SFCs expressed VCAM-1, according to immunofluorescent staining (Figs. 1D and 2D). Flow cytometric analysis showed that more than 95% of *ex vivo*-expanded SFMSCs and SFCs expressed CD90, CD44, CD105, and CD73. Stro-1, CD146, CD45, CD34, CD11b, CD19, and HLA-DR were not detected on either cell type in our flow cytometric analysis (Fig. 4).

### Differentiation of SFMSCs and SFCs

**Figure 5:**
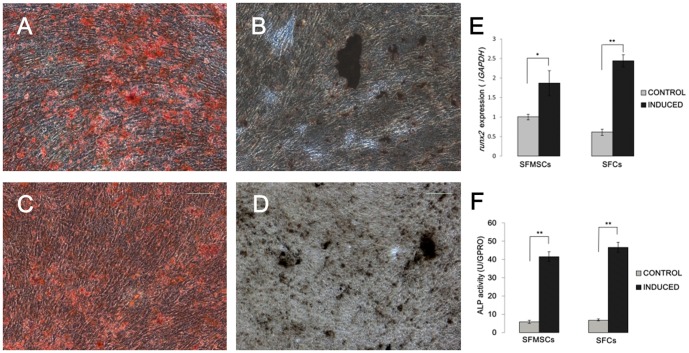
Osteogenic differentiation of SFCs and SFMSCs after culture in induction medium for 28 days. Alizarin red staining identified calcium deposits in cultured **(A)** SFCs and **(C)** SFMSCs. von Kossa staining identified calcium deposits in cultured **(B)** SFCs and **(D)** SFMSCs. Scale bars  =  100 µm. **(E)** Upregulated expression of *runx2* and **(F)** ALP activity in SFCs and SFMSCs after osteogenic induction compared to that in control cells. Significant differences between the two groups are indicated by single and double asterisks (*p<0.05, **p<0.01).

Osteogenic differentiation. After culture in osteogenic induction medium for 28 days, calcium deposits were observed in both SFMSC and SFC cultures and confirmed by von Kossa and Alizarin red staining (Fig. 5A-D). Expression of *ocn*, which is a marker for late stage matrix-producing osteoblasts, was not detected in either cell type (data not shown), indicating that the cells did not progress completely to mature osteoblasts under these culture conditions. However, after culture in osteogenic induction medium for only 14 days, *runx-2* expression and ALP activity were upregulated in both cell types (Fig. 5E, F). The expression of osteogenic transcription factor gene *runx-2* was 1.9- and 4.0-fold higher in SFMSCs and SFCs, respectively, than that in the control group after osteogenic induction for 14 days (Fig. 5E). The expression levels of ALP, an enzyme known to be upregulated during osteogenesis, in SFMSCs and SFCs were 7.1- and 6.9-fold higher, respectively, than that in the control group after osteogenic induction for 14 days (Fig. 5F).

#### Adipogenic differentiation

**Figure 6:**
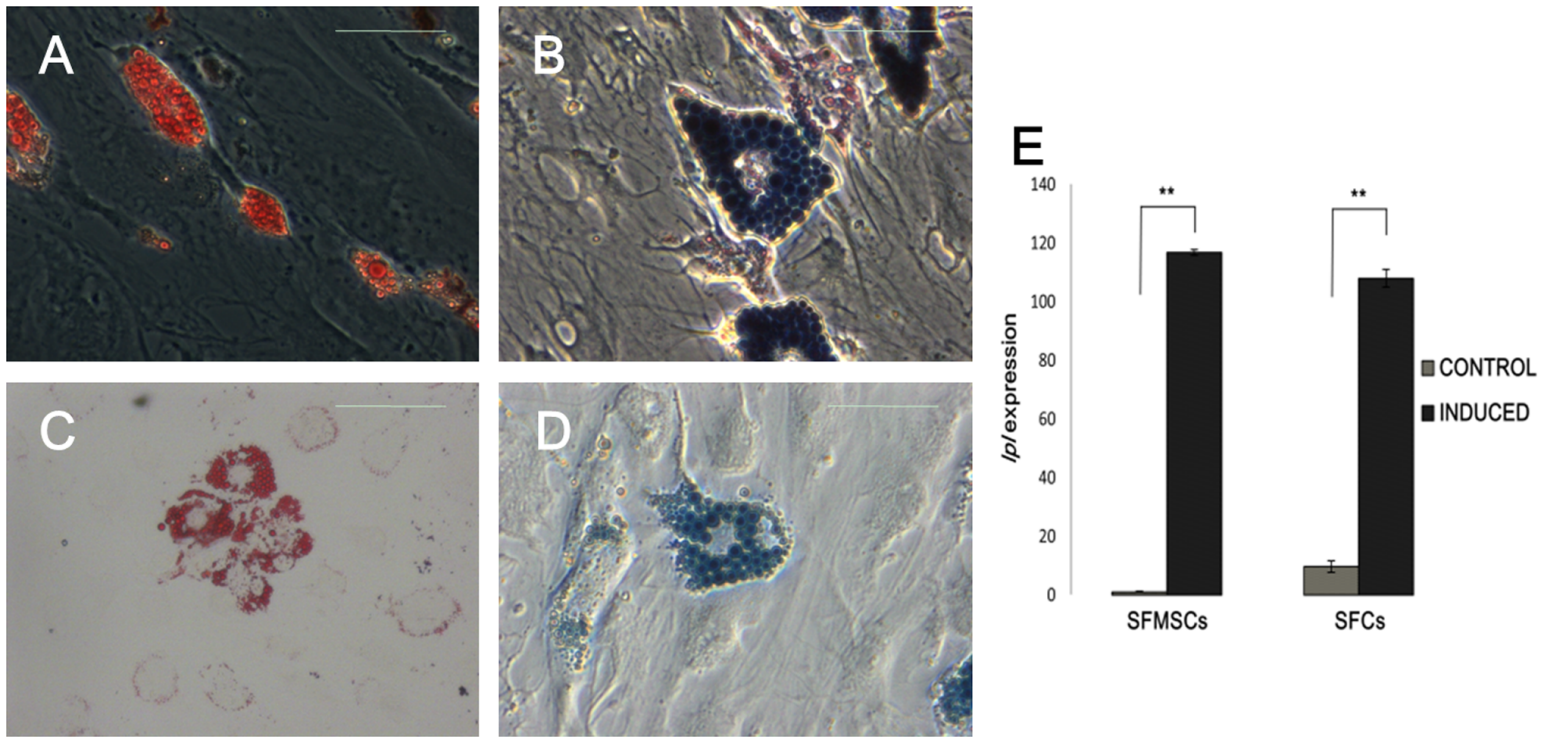
Adipogenic differentiation of SFCs and SFMSCs after culture in induction medium for 28 days. Lipid droplets were observed in SFCs after the induction period by **(A)** oil red O staining and **(B)** Sudan black B staining. Lipid droplets were also observed in SFMSCs after the induction period by **(C)** oil red O staining and **(D)** Sudan black B staining. Scale bars  =  100 µm. **(E)** After culture in induction medium for 28 days, *lpl* expression was upregulated in both SFCs and SFMSCs compared to the respective control cells. Significant differences between the two groups are indicated by single and double asterisks (*p<0.05, **p<0.01).

After 28 days of culture in adipogenic induction medium, both SFMSCs and SFCs developed into oil red O-positive and Sudan black B-positive, lipid-laden fat cells (Fig. 6A-D). In addition, *lpl* expression in SFMSCs and SFCs was significantly up-regulated at day 28 of induction, whereas both control groups expressed negligible levels of *lpl* (Fig. 6E). Upregulation of *lpl,* a lipid exchange enzyme gene known to be upregulated during adipogenesis, further confirmed the adipogenic differentiation ability of both cell types.

#### Chondrogenic differentiation

**Figure 7:**
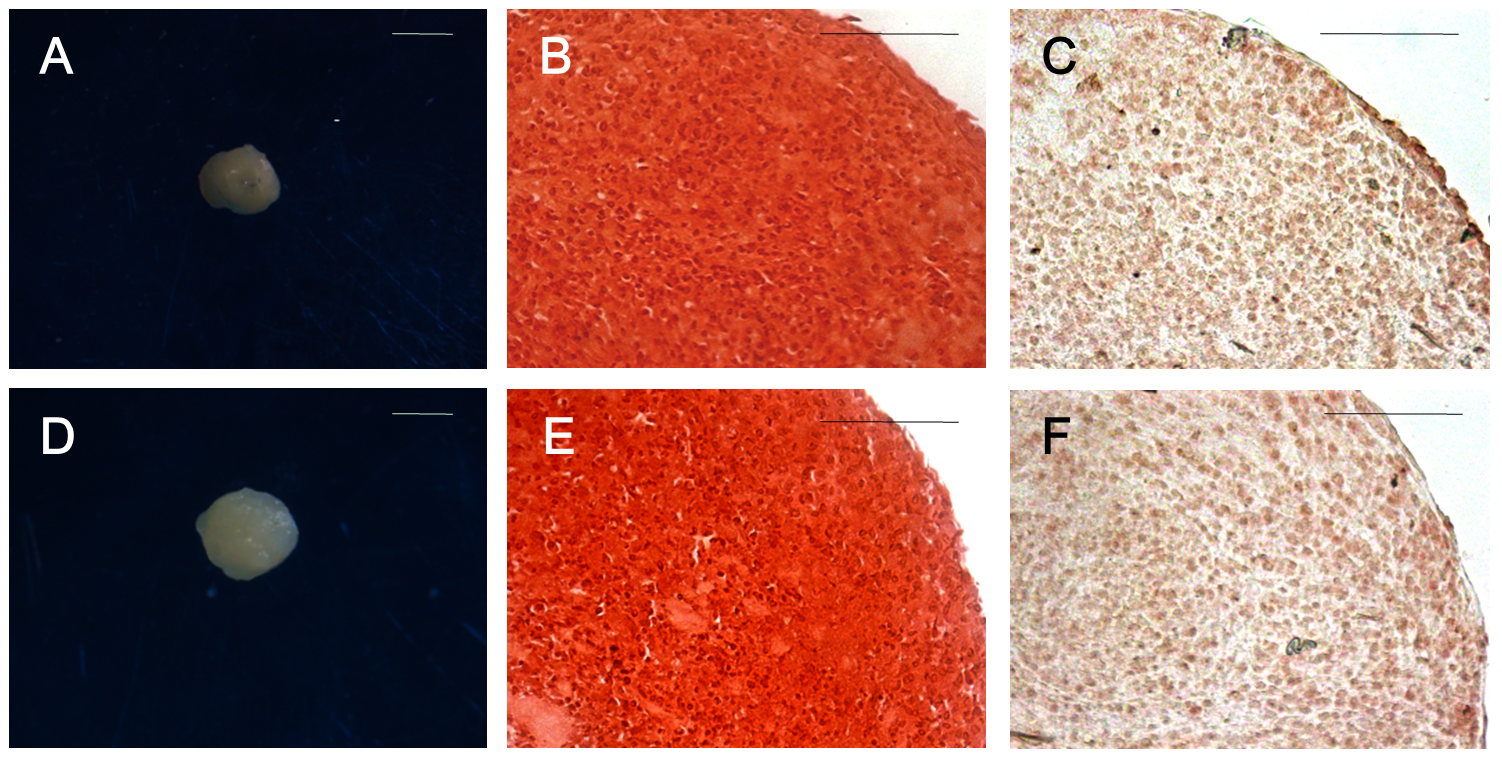
Chondrogenic differentiation of SFCs and SFMSCs after culture in induction medium for 21 days. (A) Cartilage nodule formed by SFCs after the induction period. **(B)** Safranin O staining and **(C)** collagen type II immunohistochemical staining of cartilage nodules formed by SFCs. **(D)** Cartilage nodule formed by SFMSCs after the induction period. **(E)** Safranin O staining and **(F)** collagen type II immunohistochemical staining of cartilage nodules formed by SFMSCs. Scale bars  =  1 mm in **(A, D)** and 100 µm in **(B, C, E, F)**.

After 21 days of culture in chondrogenic induction medium, both SFMSCs and SFCs formed cartilage nodules (Fig. 7A, D), and this was not observed in the respective control cell cultures. Histological sections of the cartilage nodules formed by both cell types were positively stained with Safranin O (Fig. 7B, E). In addition, immunochemical staining for collagen type II, a characteristic collagen type of cartilage, was positive throughout sections of nodules formed by both cell types (Fig. 7C, F), whereas no immunostaining was detected in the respective control cells.

#### Neurogenic differentiation

**Figure 8:**
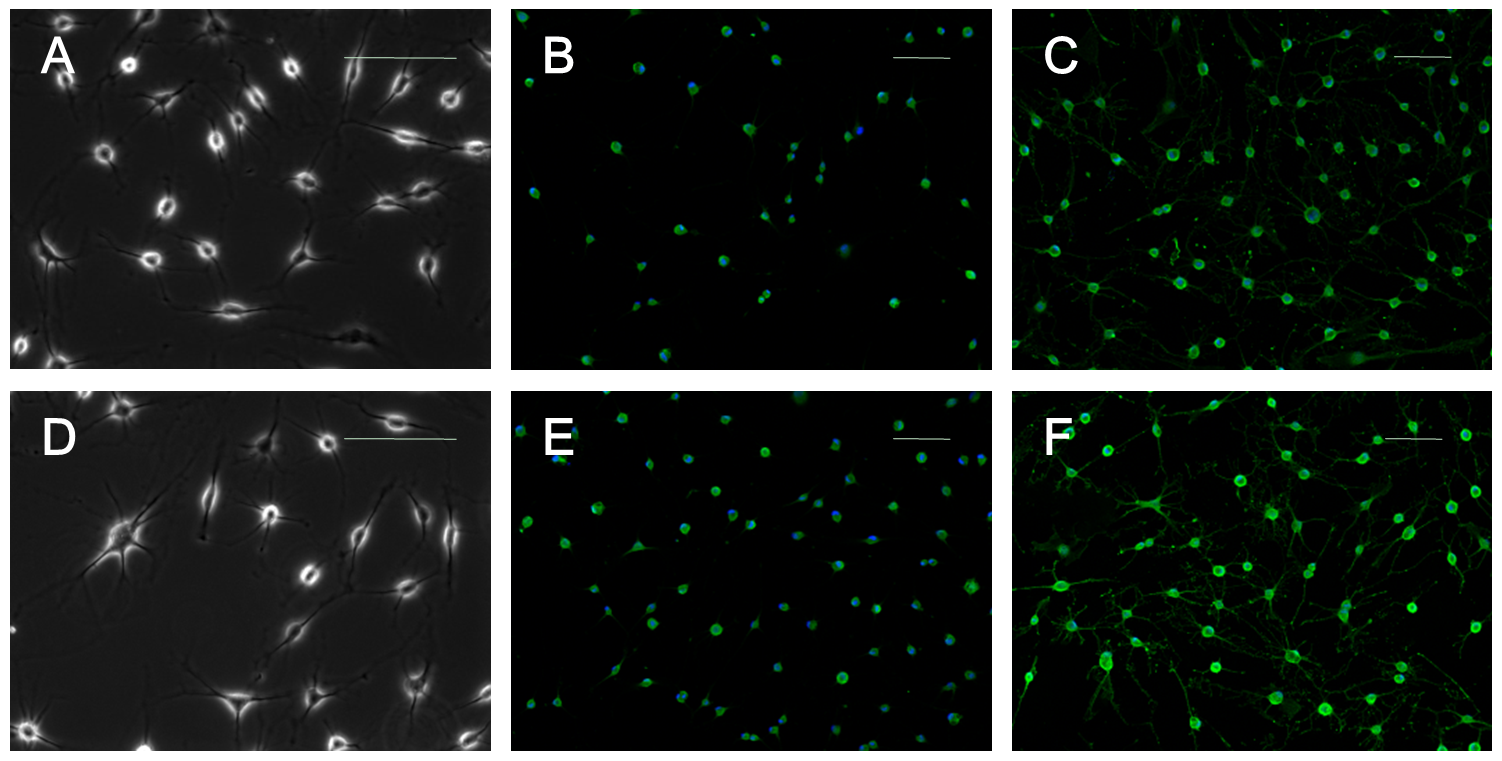
Neurogenic differentiation of SFCs and SFMSCs after culture in induction medium for 12 hours. (A) SFCs acquired a bipolar and stellate morphology by the end of the induction period and stained positively for **(B)** nestin expression and **(C)** glial fibrillary acidic protein expression. **(D)** SFMSCs also acquired a bipolar and stellate morphology by the end of the induction period and stained positively for **(E)** nestin expression and **(F)** glial fibrillary acidic protein expression. Scale bars  =  100 µm.

After 12 hours of culture in neurogenic induction medium, both SFMSCs and SFCs exhibited a bipolar and stellate morphology (Fig. 8A, D). The expression of neuron-associated markers nestin and glial fibrillary acidic protein (a specific marker for the astrocyte lineage) in SFMSCs and SFCs was demonstrated by immunofluorescent staining (Fig. 8B, C, E, F). No immunostaining was detected in the respective control cells.

## Discussion

MSCs are highly clonogenic cell s capable of both self-renewal and differentiation down multiple lineages[19]. The Mesenchymal and Tissue Stem Cell Committee of the International Society for Cellular Therapy (ISCT)[20] proposed minimal criteria for use as the uniform definition of human MSCs. First, MSCs must be adherent to plastic when expanding in standard culture condition. Second, MSCs must express CD90 (an endoglin originally recognized by the MAb SH2), CD105 (an ecto-5'-nucleotidase originally recognized by the MAb SH3 and SH4), and CD73 (known as Thy-1), and lack expression of CD34 (a primitive hematopoietic progenitor and endothelial cell marker), CD45 (a pan-leukocyte marker), CD79alpha or CD19 (a B cell marker), CD14 or CD11b (prominently expressed on monocytes and macrophages), and HLA-DR (a marker only present in MSCs upon with stimulation with interferon-γ). Third, MSCs must show multipotent differentiation potential. The phenotype characteristics of SFMSCs and SFCs in our present study meet these ISCT criteria. Koyama et al.[11] previously reported the existence of CD146^+^STRO-1^+^CD34^-^CD45^-^ SFMSCs in synovial fluid samples of patients with TMDs obtained through arthroscopy. In our present study, we complimentarily demonstrated additional surface markers in SFMSCs. However, we demonstrated that SFMSCs derived from synovial fluid removed from the TMJ of TMD patients via arthrocentesis were almost completely Stro-1 and CD146 negative. To some extent, our conclusion is inconsistent with that of the previous report by Koyama et al. [11], in which it was reported that SFMSCs expressed STRO-1 and CD146 based on immunofluorescence assays and flow cytometry. However, only immunofluorescence results for STRO-1 expression were displayed in the paper, and information about their other results as well as the percentage of STRO-1+CD146+ cells among SFMSCs were not provided. Thus, the differences between these cells in their study were unclear. Additionally, there is still a lack of consensus concerning the value of STRO-1 and CD146 as definitive markers of MSCs, because the frequency of STRO-1 and CD146 expression in MSCs is not stable and highly depends on donor, culture conditions, and cell status[21]–[23]. Simmons et al.[24] reported that STRO-1 antigen is progressively lost during culture. Russell et al.[23] discovered that surface expression of CD146 is closely associated with proliferation status. Zuk et al.[25] reported that the frequency of STRO-1 expression in adipose-derived MSCs is 31%, whereas Gronthos et al.[26] reported STRO-1 antigen was not detected in adipose-derived MSCs[23]. Hung et al.[27] reported that BMSCs are STRO-1 negative. In addition, Sekiya et al.[14] reported that SFMSCs derived from the knee joint are almost CD146 negative. Based on these conflicting reports, we speculate that the different frequencies of STRO-1 and CD146 expression in SFMSCs between our present study and that of Koyama et al. may be due to differences in culture conditions, donors, or cell status.

Next, we confirmed that the Stro-1^-^CD146^-^ SFMSCs could be induced to undergo osteogenic, adipogenic, chondrogenic, and neurogenic differentiation *in vitro*. In addition, these SFMSCs exhibited a high self-renewal capacity, allowing for the generation of a large number of cells via the *in vitro* expansion of only a small number of primary cells. Furthermore, the diluted synovial fluid specimens from which the cells were isolated were a by-product of TMJ arthrocentesis treatment performed under local anesthesia, making these cells an easily accessible resource that would likely be well accepted by patients relative to cells that require more invasive collection procedures. Based on the characteristics described above, SFMSCs may be a promising cell source for tissue engineering applications.

The tissue origin of SFMSCs remains unclear at present. Jones et al.[9] proposed these cells may be derived from disrupted cartilage, bone, synovium, periosteum, or bone marrow. In our study, we were able to obtain SFMSCs via arthrocentesis of the upper compartment of the TMJ from TMD patients who did not have perforation in the joint disk (data not show). This suggests that disrupted articular cartilage or bone may not be the tissue origin of these SFMSCs in the TMJ (at least not the only origin). The fraction of CD106^+^ cells in primary cultured bone marrow-derived MSCs varies considerably among donors and is reduced with increased passage number during *in vitro* expansion[28]. However, in our study, most SFMSCs from the TMJ of almost all donors were VCAM-1^+^ and did not show a reduction in VCAM-1 expression during *in vitro* expansion for up to 8 passages (data not shown). Furthermore, Morito et al.[10] compared the SFMSCs numbers in knee reconstruction patients undergoing anterior cruciate ligament (ACL) repair on days 1 and 6 postoperatively to evaluate whether bone marrow-derived or circulating MSCs might be the origin of such cells. Their results indicate that SFMSCs originate neither from the bone marrow nor from circulating MSCs.

Synovial tissue obtained by operation or arthroscopy usually consists of two anatomically distinct layers: intima and subintima. The intima is loosely organized, avascular, and not supported by a basement membrane, and is most often referred to as the synovium. The subintima consists of a network of connective tissue interspersed with cells and blood vessels. Fibroblast-like synoviocytes (FLSs) reside in both normal intima and subintima[29]. To date, several studies have confirmed that a portion of these FLSs exhibit multipotent capabilities under specific culture conditions[13], [30]–[32]. However, without a marker specific to these synovial membrane-derived MSCs (SMMSCs), the spatial distribution of SMMSCs within the synovial tissue cannot be elucidated. De Bari et al.[30] speculated that precursor cells from the circulation, bone marrow, or vascular pericytes might be the origin of SMMSCs, based on the bilayer anatomical structure of the synovial tissue.

In our study, the SFs found in synovial fluid collected from some TMD patients were likely derived from disrupted intima, according to their physical properties on visual and histological evaluation. Harvanova et al.[13] reported that the percentage of CD105^+^ cells among the MSC population from SM before immunomagnetic separation was between 40–50% and could be increased to 95% via immunomagnetic separation. However, our data showed that the percentage of CD105^+^ cells isolated from the SFs was approximately 95% without immunomagnetic separation. This result may be due to the different synovial tissue origin. Whereas the synovial tissue obtained via operation or arthroscopy consists of intima and subintima, the SFs obtained in our study were likely derived from the intima only. As such, these specimens may play a valuable role in the study of the spatial distribution of SMMSCs and the tissue origin of SFMSCs in the TMJ. For quite a long time, SMMSCs were believed to reside in both the intima and subintima of synovial tissue. In contrast, our study offers clear evidence supporting the notion that SFMSCs of the TMJ exist primarily in the intima of the TMJ.

Our data also show that almost all SFCs collected in this study expressed VCAM-1, like SFMSCs. VCAM-1 is one marker of a specialized phenotype of FLSs in the intima[29]; the constitutive expression of VCAM-1 by FLSs is unusual. Follicular dendritic reticulum cells in lymphoid tissue also constitutively express VCAM-1, as does a fraction of vascular wall cells and BMSCs, but the majority of fibroblastic cells do not[33]. Furthermore, the multipotent differentiation ability and phenotype of the SFCs were similar to those of SFMSCs. We also found that in primary cultures of SFMSCs *in vitro*, some cell clones grew out of small cell masses, which seemed to have broken off from the intima. This interesting phenomenon has not been commonly observed, but it confirms the speculation that the intima may shed cells in the articular cavity under a pathological state. All of the findings described above suggest that the disrupted intima is the most likely tissue origin of SFMSCs in the TMJ. This conclusion further supports the speculation of Koyama et al.[11] Koyama et al.[11] speculated that when disc injury causes rupture and bleeding, vessel injury and bleeding promote the expression of chemokines and cytokines, resulting in the recruitment of MSCs, which is in accordance with Morito et al.'s speculation[10]. Even so, they still assumed SFMSCs were possibly derived from dislodged SFs. In addition, Morito et al.[10] also assumed that SFMSCs in the knee joint may be derived from the synovium, because the morphology, gene expression profiles, and colony size of SFMSCs seemed to be more similar to those of SMMSCs than to those of BMSCs. Furthermore, joint injury is also likely to cause intima disruption and consequently trigger an increase in SFMSCs.

Lee et al.[8] reported that the levels of CD34^-^CD44^+^CD90^+^ SFMSCs in the knee are associated with the severity of primary knee osteoarthritis. Morito et al.[10] reported that SFMSCs in the knee increase after intra-articular ligament injury in humans. This is particularly noteworthy, and to some extent, SFMSCs originating from disrupted intima can explain these clinical features. Furthermore, the levels of SFMSCs in the TMJ also are likely to be closely associated with the severity of TMDs based on our results. Further clinical studies are needed to confirm this speculation. If this is in fact the case, the level of SFMSCs may become a new reference index for the diagnosis of TMDs in the future.

The differentiation potential of SFMSCs isolated from the TMJ was similar to that of BMSCs[11]. Several studies have demonstrated the therapeutic utility of BMSCs in human joint diseases[4], [34]–[37]. However, to our knowledge, the therapeutic utility of SFMSCs has been rarely reported, potentially because the roles of SFMSCs in joints remain unclear. The behavior of SFMSCs in the TMJ of patients with TMDs may be a benefit to the repair processes. Jones et al.[38] reported that SFMSC levels in normal human knee joints increase 7-fold during the early stages of osteoarthritis. They speculated that this increase in the number of SFMSCs represents an effort to restore homeostasis in the joint. It is now well-accepted that tissue destruction in OA is closely associated with attempted repair processes[11], [39]. However, Neidhart et al.[40] reported that synovial fluid-derived FLSs mediate cartilage destruction in rheumatoid arthritis. Thus, further study is needed to determine whether SFMSCs are released into the joint cavity to support the reconstruction of articular cartilage or to participate in the pathogenic processes of arthritis.

## Conclusion

To our knowledge, the present study offers the first evidence that SFMSCs obtained during TMJ arthrocentesis are almost completely Stro-1 and CD146 negative but do meet the ISCT standards for MSCs. Although we were unable to definitely determine the tissue source of SFMSCs in the TMJ, our results suggest that disrupted intima is the most likely tissue origin of these cells. Unlike synovial specimens obtained during surgery, the SFs collected in this study consisted only of intima and thus may represent a better cell source for the study of synovium-derived MSCs.
